# Retraction Note: MAGE-C2/CT10 promotes growth and metastasis through upregulating c-Myc expression in prostate cancer

**DOI:** 10.1007/s11010-023-04820-1

**Published:** 2023-07-27

**Authors:** Jun Qiu, Bei Yang

**Affiliations:** 1 Center of Clinical Laboratory, The First Affiliated Hospital of Soochow University, No. 899 Pinghai Road, Suzhou, 215000 Jiangsu China; 2 Department of Imaging, Tongji Medical College, The Central Hospital of Wuhan, Huazhong University of Science and Technology, No. 26 Shengli Street, Jiangan District, Wuhan, 430014 Hubei China

**Molecular and Cellular Biochemistry (2020) 476:1-10**

10.1007/s11010-020-03814-7

The authors have retracted this article because, after publication, it was brought to their attention that some images overlap with those in other articles. In particular:

- Figure 7B (panel 400X vs. Ad-shNC) overlaps with Fig. 7D (panel Ki67 vs. Ad-shNC) of [1]
- Figure 7B (panel 100X vs. Ad-snNC) overlap with Fig. 2F MCF-7 (panel 100X vs. NC) of [2]

The authors have stated that the immunohistochemistry experiments were performed by a third party; this was not reported in the article. All authors agree to this retraction.

## References

[1] Zhou K, Mai H, Zheng S, Cai W, Yang X, Chen Z, Zhan B (2020). OTUB1-mediated deubiquitination of FOXM1 up-regulates ECT-2 to promote tumor progression in renal cell carcinoma. Cell & bioscience.

[2] Dong L, Chen F, Fan Y, Long J (2020). MiR-34b-5p inhibits cell proliferation, migration and invasion through targeting ARHGAP1 in breast cancer. Am J Translational Res.

